# Characterization of Nucleotide Misincorporation Patterns in the Iceman's Mitochondrial DNA

**DOI:** 10.1371/journal.pone.0008629

**Published:** 2010-01-08

**Authors:** Cristina Olivieri, Luca Ermini, Ermanno Rizzi, Giorgio Corti, Raoul Bonnal, Stefania Luciani, Isolina Marota, Gianluca De Bellis, Franco Rollo

**Affiliations:** 1 Laboratorio di Archeo-Antropologia molecolare/DNA Antico, Dipartimento di Biologia Molecolare, Cellulare e Animale, University of Camerino, Camerino, Italy; 2 Institute of Cellular Medicine, University of Newcastle, Newcastle, United Kingdom; 3 Istituto di Tecnologie Biomediche, Consiglio Nazionale delle Ricerche, Milano, Italy; Niels Bohr Institute and Biological Institutes, Denmark

## Abstract

**Background:**

The degradation of DNA represents one of the main issues in the genetic analysis of archeological specimens. In the recent years, a particular kind of post-mortem DNA modification giving rise to nucleotide misincorporation (“miscoding lesions”) has been the object of extensive investigations.

**Methodology/Principal Findings:**

To improve our knowledge regarding the nature and incidence of ancient DNA nucleotide misincorporations, we have utilized 6,859 (629,975 bp) mitochondrial (mt) DNA sequences obtained from the 5,350–5,100-years-old, freeze-desiccated human mummy popularly known as the Tyrolean Iceman or Ötzi. To generate the sequences, we have applied a mixed PCR/pyrosequencing procedure allowing one to obtain a particularly high sequence coverage. As a control, we have produced further 8,982 (805,155 bp) mtDNA sequences from a contemporary specimen using the same system and starting from the same template copy number of the ancient sample. From the analysis of the nucleotide misincorporation rate in ancient, modern, and putative contaminant sequences, we observed that the rate of misincorporation is significantly lower in modern and putative contaminant sequence datasets than in ancient sequences. In contrast, type 2 transitions represent the vast majority (85%) of the observed nucleotide misincorporations in ancient sequences.

**Conclusions/Significance:**

This study provides a further contribution to the knowledge of nucleotide misincorporation patterns in DNA sequences obtained from freeze-preserved archeological specimens. In the Iceman system, ancient sequences can be clearly distinguished from contaminants on the basis of nucleotide misincorporation rates. This observation confirms a previous identification of the ancient mummy sequences made on a purely phylogenetical basis. The present investigation provides further indication that the majority of ancient DNA damage is reflected by type 2 (cytosine→thymine/guanine→adenine) transitions and that type 1 transitions are essentially PCR artifacts.

## Introduction

Within living cells, the integrity of DNA molecules is continually maintained by enzymatic repair processes [1]. After the death of an organism, cellular compartments that normally sequester catabolic enzymes break down and, as a consequence, DNA is rapidly degraded by cellular enzymes. A further source of degradation is represented by bacteria, fungi, and soil invertebrates that, overtime, feed on and degrade macromolecules [2]. According to the studies of Tomas Lindahl [1], spontaneous chemical reactions can arise and lead to a partial or total degradation of the DNA molecule. These studies have shown that, for DNA in aqueous solution, hydrolytic cleavage of the base-sugar bond (N-glycosidic bond) leads to the loss of nucleotidic bases and induces the formation of apurinic/apyrimidinic (AP) sites [1]. These baseless sites strongly destabilize the DNA structure and, consequently, strands of the double helix are broken down by a β-elimination reaction [3]. As time goes on, this mechanism leads to a progressive fragmentation of the whole molecule into tiny fragments. Hydrolysis is also responsible for base deamination reactions which produce damage in ancient DNA templates. Cytosine (C) and its homologue 5-methylcytosine are the main targets for the hydrolytic deamination and as a result of this reaction the two bases are converted to either uracil (U) or thymine (T), respectively [1]. In the experimental system used by Lindahl, deamination of DNA purines such as adenine (A) and guanine (G) is less frequent (deamination rate of C is ∼30–50 times higher than that of A; [1]) and generates hypoxanthine (H) and xanthine (X) from adenine and guanine respectively.

Another chemical reaction strongly involved in DNA degradation over time is oxidative damage which destroys the ring structure of nucleotidic bases and leads to strand breakage.

Subsequent studies to those of Tomas Lindahl in which DNA was isolated from ancient specimens have shown a further type of post-mortem DNA modifications. There are interstrand crosslinks [4] and cross-linking of DNA to other biomolecules including RNA, proteins, sugars, and fatty acids [5], [6]. Other mechanisms, such as alkylation or UV irradiation, are unlikely to affect buried remains. [1].

Despite the fact that most human diploid cells contain several billion bases of nuclear DNA, and thousands of mitochondrial DNA copies, DNA decay may be so fast that, even in a short period, no or few PCR-amplifiable templates can remain [1]. As a consequence, most ancient specimens either do not contain any amplifiable endogenous DNA or contain only low molecular weight endogenous DNA within a size range of 100–500 base pair (bp) [7]–[9].

Under rare circumstances, such as rapid tissue desiccation after death, or when DNA is adsorbed in a mineral matrix, or when a corpse is preserved at low temperatures, nucleic acids may escape enzymatic and microbial degradation, and several copies can still be found after a prolonged period of time [10].

DNA degradation and the resulting lesions represents a major issue in the genetic analysis of ancient samples. DNA lesions such as baseless sites, strand breaks and crosslinks block the extension of polymerase enzymes, thus rendering the molecules unsuitable as templates for PCR. Others, termed miscoding lesions, allow for amplification, but result in the incorporation of erroneous bases during PCR [1], [6], [7], [9]–[16].

A further issue in the analysis of DNA isolated from ancient specimens is the fact that DNA polymerase itself can be a source of misincorporations. DNA synthesis by a DNA polymerase is a highly ordered and complex molecular process. As a consequence, the enzyme can introduce impaired nucleotides with a certain frequency. This phenomenon is currently referred to as polymerase error. Error rates in PCR vary according to the precise DNA sequence and the in vitro conditions of DNA synthesis. In the case of PCR catalyzed by the thermostable *Thermus aquaticus* (Taq) DNA polymerase the observed error frequency vary from approximately 2×10^−4^ to 1×10^−5^ [17].

Nucleotide misincorporations and their possible origin (either miscoding lesions or polymerase error) have been the object of a number of investigations [9]–[13], [18]–[23].

According to the literature [10], [11], [13], the most frequent nucleotide misincorporations are the four transitions A→G, C→T, G→A and T→C, and, due to the complementary nature of DNA strands, these transitions can be grouped into two different classes termed type 1 (A→G/T→C) and type 2 (C→T/G→A) transitions.

Although type 1 and type 2 transitions have been observed among virtually all ancient DNA (aDNA) datasets, a controversy has concerned which type of transition represents true post-mortem damage and which type of transition is an artefact of regular DNA polymerase errors.

Furthermore different studies have shown different ratios of type 1 and type 2 transitions.

Gilbert et al. [13], analysing miscoding lesions in ancient human mitochondrial DNA, observed ∼31% type 1 and ∼62% type 2 transitions (type 1 to type 2 ratio of 1∶2) and ∼7% transversions. Binladen et al. [18] compared miscoding lesion damage in mitochondrial and nuclear ancient DNA and noticed ∼23% type 1, ∼56% type 2 (type 1 to type 2 ratio of 1∶2) and ∼20% transversions. Both studies, hence, show a bias toward type 2 and demonstrate a type 1 to type 2 ratio close to ∼1: 2. On the other hand, Hofreiter et al. [10] found almost exclusively type 2 transitions in mitochondrial sequences from Pleistocene cave bears.

It is important to emphasise that all these studies were performed using PCR amplification followed by Sanger methodology sequencing.

In 2006 Stiller et al. [19] by repeated amplifications of mitochondrial DNA sequences from a large number of ancient wolf remains, showed that C→T/G→A transitions are the predominant type of nucleotide misincorporations which are caused mainly by modifications of C residues. A year later Brotherton et al. [21], applying a SPEX-based approach on ancient DNA sequences retrieved from three mammalian species (bison, human, Eurasian cave-lion), provided strong quantitative evidence that substitution of C to U is the major cause of miscoding lesions. Gilbert et al. [22], using pyrosequencing technology and comparative analysis of modern chloroplast DNA (390,965 bp) and permafrost-preserved ancient woolly mammoth DNA (131,474 bp), showed that type 2 miscoding lesions represent the overwhelming majority (88% total miscoding lesions, 94% of transitions) of damage with a type 1 to type 2 ratio of about 1∶15.

Briggs et al. [20] analysing ancient genome sequences from a Neandertal, a mammoth, and a cave bear, generated by high-throughput direct sequencing techniques provided further evidence that miscoding cytosine residues are vastly overrepresented in ancient DNA sequences. The authors also showed that miscoding lesions are clustered in the ends of the molecules, and that purines are overrepresented at positions adjacent to the breaks. This last finding, therefore, suggests that depurination contributed to DNA degradation overtime.

Ermini et al. [24], using a mixed sequencing procedure based on PCR amplification and pyrosequencing of pooled amplification products, obtained the complete mitochondrial genome sequence of a prehistoric human mummy dating 5,350–5,100 years before present [25]. This mummy (popularly known as the Iceman, Similaun Man, or Ötzi), was discovered on 19^th^ September 1991, at 3,270 m above sea level in the Eastern Alps near the Austro-Italian border. It is believed to be the result of a spontaneous freeze-drying process. Since the discovery, the mummy has been preserved at temperatures below 0°C.

To refine our knowledge on nucleotide misincorporation patterns in ancient DNA sequences, we have used 6,916 sequences of the Iceman's mitochondrial DNA generated with a mixed PCR–pyrosequencing procedure [24] and compared them with 8,982 sequences of modern human mtDNA generated specifically for use in the present study.

The use of a human system for the analysis of misincorporation patterns might raise concern because of the contamination issue compared to an animal or plant system. The Iceman's mtDNA system, however, provides a particular guarantee of reliability due to (a) the state of preservation of the mummy's DNA and (b) the detailed examinations of the putative contaminants previously performed [24].

## Materials and Methods

### Ethics Statement

The modern DNA utilized in this work was provided by one of the authors.

### DNA Sequences from the Mummy

Mitochondrial DNA sequences from the Iceman were obtained as reported by Ermini et al. [24]. Briefly, DNA extracted from the mummy's intestinal content was PCR amplified using a set of 235 primer pairs. Amplification products were pooled and used as a substrate for the pyrosequencing reaction [26]. The GS-FLX genome sequencer (FLX Roche 454 LifeSciences) yielded a total of 45,829 mtDNA short sequences clustered in 235 clonal groups [24]. In the present paper, the characterization of the miscoding lesions pattern was performed on a total of 44 clonal groups of sequences (6,916 sequences) that had been previously shown to contain polymorphisms specific to the Iceman. They had previously been subdivided into two groups of 6,719 (617,281 bp) and 197 (26,048 bp) sequences (corresponding to original and putative contaminant sequences respectively) on a phylogenetical basis [24].

In order to ensure an adequate level of sequence coverage we discarded all clonal groups of sequences showing a coverage <10. As a result, we finally utilized 6,719 sequences from the first group and 140 (12,694 bp) sequences from the second.

### DNA Sequences from a Modern Specimen

Cell samples were taken by scraping the buccal mucus of an author of the paper (mitochondrial haplogroup V) with a conical brush. DNA was extracted by means of a phenol/chloroform protocol [27].

To determine the mitochondrial DNA copy number fragment in the preparation we performed a quantitative PCR (qPCR), as described in Ermini et al. [24].

We carried out 44 PCR amplifications using as template the same amount of DNA as used in the ancient DNA amplification i.e. approximately 5,000–6,000 copies of mtDNA per reaction tube. The 44 PCR amplications were performed using the same primers, reaction mix and thermal profile described by Ermini et al. [24]. Amplification products were checked by electrophoresis, then purified using a High Pure PCR Product purification kit (Roche Molecular Biochemicals, Indianapolis, IN). The amplification products were diluted to equal concentrations, then pooled in equimolar proportion and used as a substrate for the pyrosequencing reaction [24], [26].

The GS-FLX genome sequencer yielded a total of 9,489 mtDNA sequences. The sequences were then processed by identifying those displaying the PCR primers at their termini and eliminating those lacking them. The sequences obtained from this preliminary screening were analysed to identify the presence of nuclear mitochondrial DNA sequences (numts) that were eliminated from the modern dataset. All the sequences that showed more than one difference from the mtDNA consensus sequence were compared with the reference sequences in GenBank using the National Centre for Biotechnology Information (NCBI) BLAST search. The response was used to discriminate between nuclear and mitochondrial DNA. This screening yielded 8,982 sequences (∼94% of the total; 805,155 bp), that represent the modern DNA sequence data.

This part of the work was performed several months after the ancient DNA results had been published and in a different laboratory from that used for the manipulation of the ancient specimens.

### Nucleotide Misincorporation Analysis

Ancient, modern and putative contaminant sequences were aligned with the revised Cambridge Reference Sequence (rCRS) [28] using BioEdit Sequence Alignment Editor v. 7.0.9.0 [29]. The consensus sequence for each group of clones was determined from the shared bases and the remaining interclone base differences were attributed to either post-mortem damage (miscoding lesions) or polymerase error. In the event of contaminant sequences, the interclone base differences that were found to occur repeatedly were considered to belong to genuine mtDNA polymorphisms and therefore not considered in the analysis; insertions and deletions were excluded from our analysis. By convention, all sequences are described in the L-strand orientation.

The error rates for Hot Start *Taq* DNA polymerase (Qiagen, Hilden, Germany) and Platinum *Taq* Hifidelity polymerase (Invitrogen, Carlsbad, CA), used for the PCR amplification and for the emulsion-based clonal amplification (emPCR) in the pyrosequencing procedure, were assumed to be 2×10^−4^−1×10^−5^ (Hot Start *Taq*) and 2×10^−6^ (Platinum *Taq*) respectively as reported in the literature [17], [22], [30].

For each set of clones, a nucleotide misincorporation rate, *m*, (the probability of observing a transition or transversion in a single position of the sequence) was calculated using the formula: *m* = M/Ln; where M is the number of nucleotide misincorporations observed in each group of clones analysed, L is the length of each amplified mitochondrial fragment and n is the number of clones sequenced per each mitochondrial fragment.

Two-Sample T-Tests and non-parametric Mann-Whitney tests were performed, all at the 1% significance level, to compare the rate of nucleotide misincorporation (*m*) between: (a) ancient and modern sequences, (b) ancient and putative contaminant sequences and (c) modern and putative contaminant sequences. The tests allowed us to accept or reject the null hypothesis (H_0_) that the *m* values of the three classes of sequences are the same. To highlight the distribution of *m* among the three classes of sequences, the rate of nucleotide misincorporation for each of them was represented using a box-plot. The statistical analysis was performed using the Minitab 15.1.0.0 software.

The number and type of nucleotide misincorporations for the different sequence datasets were assessed according to Gilbert et al. [13]. For each group of clones we measured the absolute number of each of the 12 possible base changes (A→C, A→G, A→T, C→A, C→G, C→T, G→A, G→C, G→T, T→A, T→C, and T→G) which, due to the complementary nature of DNA, were grouped into six complementary pairings (A→G/T→C, A→T/T→A, A→C/T→G, C→T/G→A, C→G/G→C, C→A/G→T). All the nucleotide misincorporation values were scaled to compensate the mitochondrial fragment nucleotide composition bias. In particular, when the nucleotide misincorporations originated from an underrepresented C and G nucleotide, their values were multiplied by the ratio A+T/G+C in the specific fragment. On the other hand, when the nucleotide misincorporations originated from an underrepresented A and T nucleotide, their values were multiplied by the ratio G+C/A+T in the specific fragment

The total number of the nucleotide misincorporation types observed within modern and ancient datasets were obtain by the sum of the nucleotide misincorporation number revealed in each group of clones. These data were scaled to balance the different number of nucleotides analysed for each sample (617,281 and 805,155 in ancient and modern sample, respectively).

Two sample T-Test and non-parametric Mann-Whitney tests were performed at the 1% significance level to assess the statistical significance of nucleotide misincorporation in ancient and modern sequences. We also plotted the nucleotide misincorporation number for each transition and transversion complementary group for ancient and modern sequences. In this case too, the statistical analysis was performed using the Minitab 15.1.0.0 software.

Due to the relatively small number (140) of putative contaminant sequences described in Ermini et al. [24] and the exiguity of the nucleotide misincorporations found in them (27 in total), this group was not submitted to a detailed analysis.

## Results

Tables S1, S2 and S3 report misincorporations observed in each clonal group of ancient, modern and contaminant sequences respectively.

We find misincorporation rates of approximately 8×10^−3^ for the ancient sequences, of approximately 2×10^−3^ for the modern sequences and of approximately 2×10^−3^ for the putative contaminant sequences. The observed misincorporation rates are higher than those expected on the basis of the error rates for Hot Start *Taq* DNA polymerase (Qiagen) and Platium *Taq* Hifidelity polymerase (Invitrogen).

**Figure 1:**
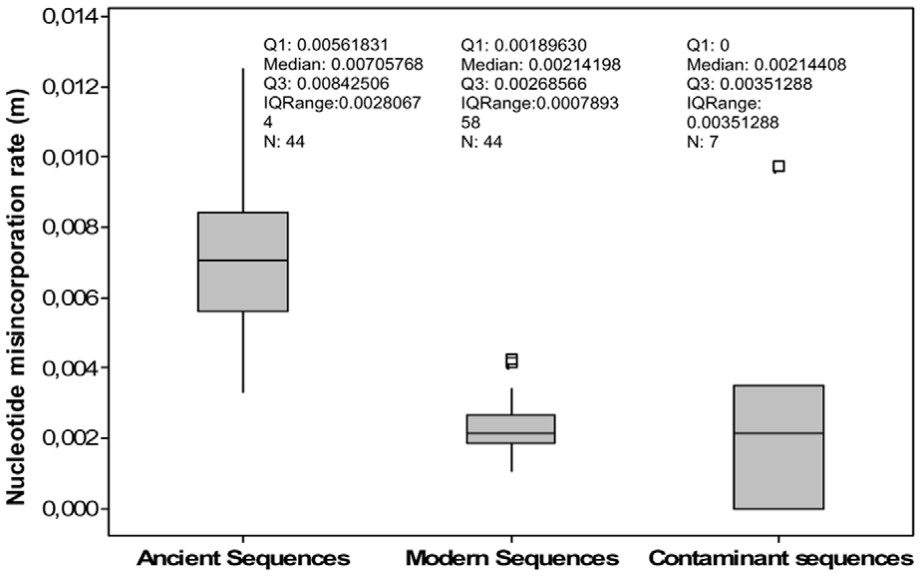
Nucleotide misincorporation rate (*m*) in ancient, modern and putative contaminant sequences. The plot comprises a box and whiskers. A line is drawn across the box to represent the median; the bottom of the box is the first quartile (Q1) and the top is the third quartile (Q3). The lower whisker extends to the lowest value within the lower limit, while the upper whisker extends to the highest value within the upper limit. The limits are defined by: Q1−1.5 (Q3−Q1) (lower limit) and Q3+1.5 (Q3−Q1) (upper limit). The square (□) represent the outlier, a value beyond the whiskers.

To compare the number of misincorporations in modern, ancient and putative contaminant sequences we calculated the rate of nucleotide misincorporation, *m*, (Tables S1, S2 and S3) scaled for the total number of nucleotides in each group of sequences. The two Sample T-Tests and non-parametric Mann-Whitney tests showed that the null hypothesis can be rejected for both the ancient and modern groups of sequences (Two sample T-test T-Value = 14.60, p-Value = 0.000; Mann-Whitney test W = 2921.0, p-Value = 0.000) and the ancient and putative contaminant groups of sequences (Two sample T-test T-Value = 3.40, P-Value = 0.010; Mann-Whitney test W = 1260.0, p-Value = 0.0016). However, the tests support the null hypothesis for the modern and putative contaminant sequences indicating that the rate of nucleotide misincorporation was the same (Two sample T-test T-Value = −0.42, P-Value = 0.688; Mann-Whitney test W = 1137.0, p-Value = 0.8588). Figure 1 displays the measure of nucleotide misincorporation in ancient, modern and putative contaminant sequences. These analyses confirm that the *m* values of ancient and modern sequences differ significantly the *m* value of the second being lower (median: 0.00214198) than that of the first (median: 0.00705768). However the putative contaminant *m* value is close to that of modern sequences.

The observed numbers of the 12 possible nucleotide misincorporations and the six complementary change groups [13] for each clonal group of ancient and modern sequences (as described in Material and Methods the putative contaminants were excluded from this type of analysis) are reported in Tables S1 and S2 respectively.

The total number of misincorporations distributed among the six complementary groups, for ancient and modern sequences are reported in Table 1. The six observed values of the complementary groups are scaled to balance the different number of nucleotides analysed for each sample (617,281 and 805,155 in ancient and modern sample, respectively).

**Table 1:** Sum of the six complementary nucleotide misincorporations observed in the ancient and modern samples.

	Nucleotide misincorporation originally derived from A and T nucleotide				Nucleotide misincorporation originally derived from C and G nucleotide			
	A→G T→C	A→T T→A	A→C T→G	Total A+T	C→T G→A	C→G G→C	C→A G→T	Total C+G
**Modern Sample**	1184.78	191.78	75.18	456,906	446.99	22.66	53.55	348,249
**Ancient Sample**	634.9	42.06	66.43	343,000	5143.16	11.02	132.33	274,281
**Corrected Ancient Sample**	845.74	56.03	88.49		6530.17	13.99	168.02	

Total A+T: Total number of adenine and thymine nucleotides analysed in modern and ancient samples. Total C+G: Total number of cytosine and guanine nucleotides analysed in modern and ancient samples. The total number of nucleotide misincorporation in modern and ancient samples are obtained by the sum of the nucleotide misincorporation values found in each ancient and modern mitochondrial fragment analysed (see Tables S1 and S2, where the nucleotide misincorporation values are scaled to compensate the local nucleotide composition bias).Corrected Ancient Sample: the values of nucleotide misincorporation among ancient sequences, scaled to match the values of total modern nucleotides analysed. For example: Corrected Ancient Sample for A→G T→C complementary group was calculated as (Ancient Sample A→G T→C)*(Total A+T in Modern Sample)/(Total A+T in Ancient Sample).

We observed that some complementary groups such as A→C/T→G, C→A/G→T transversions and C→T/G→A transitions are more frequent in the ancient dataset than in the modern, while A→T/T→A, C→G/G→C transversions and A→G/T→C transitions are more abundant in the modern dataset.

**Figure 2:**
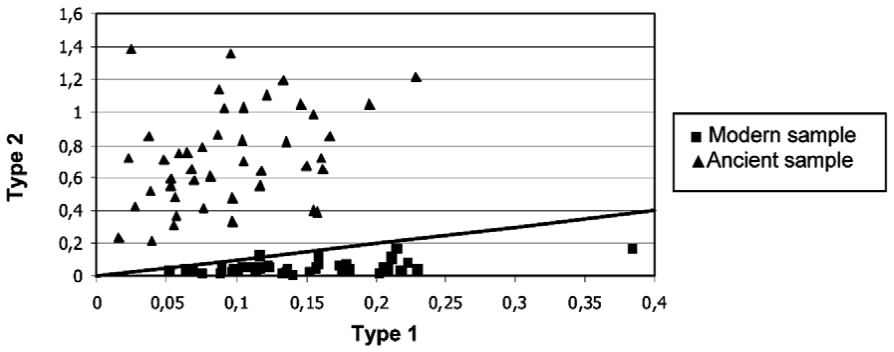
Type 1 versus type 2 transitions for modern and ancient samples. Type 1 and type 2 are standardized for a number of analysed clones for each mitochondrial fragment. The straight line represents the hypothetical situation with both transition types at a same value.

By taking into account only the nucleotide misincorporations originally derived from A and T nucleotides, we observed that type 1 (A→G/T→C) transitions are the most represented in both the modern and ancient datasets. However for the misincorporations originally derived from G and C nucleotides, the most frequent substitutions were type 2 (C→T/G→A) transitions. Considering only the ancient dataset, we calculated 845.74 type 1 and 6530.17 type 2 transitions (type 1: type 2 ratio ∼1∶8), demonstrating that type 2 transitions represent the majority (85%) of the total observed misincorporations. The modern dataset, instead, shows 1184.78 type 1 and 446.99 type 2 transitions (type 1: type 2 ratio ∼3∶1), with the majority (∼60%) of the misincorporations represented by type 1 transitions. A similar situation with a bias toward type 2 transitions for ancient sequences and a bias toward type 1 for modern was also found when individual clonal groups of sequences were considered (Figure 2).

**Figure 3:**
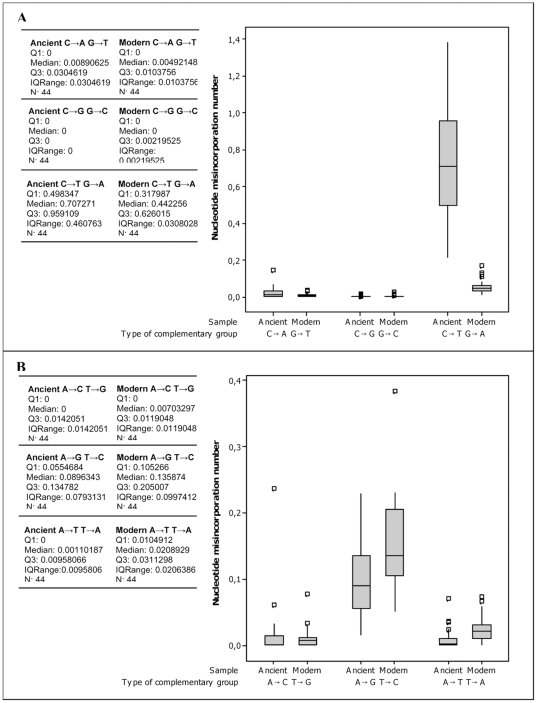
Nucleotide misincorporations number for each complementary group in the ancient and modern sequences. Nucleotide misincorporations originally derived from G and C nucleotides. (B) Nucleotide misincorporations originally derived from A and T nucleotides. The lesions number are standardized for the number of clones analysed for each mitochondrial fragment. The plot comprises a box and whiskers. A line is drawn across the box to represent the median; the bottom of the box is the first quartile (Q1) and the top is the third quartile (Q3). The lower whisker extends to the lowest value within the lower limit, while the upper whisker extends to the highest value within the upper limit. The limits are defined by: Q1−1.5 (Q3−Q1) (lower limit) and Q3+1.5 (Q3−Q1) (upper limit). The square (□) represent the outlier, a value beyond the whiskers.

The Two Sample T-Tests and non-parametric Mann-Whitney tests were performed to accept or reject the null hypothesis (H_0_) that the number of each complementary groups in the modern and ancient sequences are the same. The results of the tests are reported in Table 2 and support the null hypothesis for, A→C/T→G, C→G/G→C and C→A/G→T complementary groups. The null hypothesis was rejected for A→G/T→C, A→T/T→A and C→T/G→A complementary groups. Figure 3 displays the variance of the nucleotide misincorporation number for each complementary group in modern and ancient samples.

**Table 2:** Two Sample T-Tests and non-parametric Mann-Whitney tests between each complementary group in the modern and ancient sequences.

	Two Sample T-Test		Mann-Whitney Test	
	T-value	P-value	W	P-value
**A→G T→C**	−4.55	0.000	1458.5	0.0000
**A→T T→A**	−4.61	0.000	1360.5	0.0000
**A→C T→G**	0.61	0.546	1806.0	0.1873
**C→T G→A**	14.93	0.000	2926.0	0.0000
**C→G G→C**	−0.94	0.349	1865.5	0.2760
**C→A G→T**	2.82	0.007	2228.0	0.0215

The complementary groups A→G/T→C, A→T/T→A (Figure 3B) are more frequent in the modern than the ancient dataset, while the complementary group C→T/G→A is more frequent in the ancient than the modern dataset (Figure 3A). As a consequence, the C→T/G→A transitions plays an important role in differentiating ancient and modern sequences.

## Discussion

### The Importance of the Experimental System

Published articles on nucleotide misincorporation studies are relatively abundant in the scientific literature [9]–[13], [18]–[23]. A common weakness, however, is that most of the studies are based on not completely consistent sequence datasets. In addition one of them [23] relies on sequence data of potentially dubious authenticity, i.e. archaeological materials from Egypt. Studies on DNA degradation rates in archaeological materials from Egypt, in fact, suggest that the original DNA is not kept in these materials [31], [32].

One could add that many early studies have been conducted on a small number of clones for each ancient DNA fragment generated by Sanger technology [10], [13], [18], [23]. The development of new high-throughput sequencing techniques, such as pyrosequencing has made it possible to further characterise post-mortem damage in ancient DNA [19], [20], [22]. The usual pyrosequencing approach, however, makes use of a first step in which the whole DNA extracted from the specimen is pyrosequenced and a second step in which, among the massive high-throughput outcome, only the endogenous DNA sequences are selected. Consequently, due to the presence of microbial DNA in archaeological specimens, the sequence coverage for the original sequences can be modest; examples of this approach are given by pyrosequences obtained from Pleistocene cave bears [33] and mammoth bones [34].

To investigate the nature of nucleotide misincorporations in ancient DNA, we have considered a set of ancient sequences obtained in our laboratory, the authenticity of which has been previously ascertained [24]. We have used a new procedure based on PCR amplifications of overlapping DNA fragments followed by high throughput sequencing using 454 pyrosequencing technology [24]. This approach allows one to obtain robust sequence coverage. The Tyrolean Iceman's mtDNA, indeed, shows a coverage ranging from 19 to 1,030 reads, corresponding to a mean coverage of ∼153 and a modal coverage of 41 reads while the modern dataset displays a coverage spanning from 26 to 770 reads with a mean coverage of ∼204 and modal coverage of 52 reads. Putative contaminant sequences, are poorly represented (coverage ranging from 13 to 29 reads with a mean and modal coverage of 20 and 14 reads, respectively). This is attributable to different causes: 1) the well preserved state of the mummy and the consequent high copy number of endogenous DNA remaining; 2) the protection offered by the body (samples were taken from inside the intestines); 3) the precautions adopted during sampling and subsequent laboratory investigations [24].

A further element of reliability in our work is that the starting copy number of template molecules for ancient or modern DNA preparations is similar and also the type of DNA is the same (human mtDNA). The potential issues posed by the use of heterogeneous genetic systems were considered by previous authors [22] yet circumstances prevented them from the use of more consistent systems. Finally, similar to several previous papers [19]–[22] the large number of sequences obtained and the high coverage of each mitochondrial fragment analysed have allowed us to apply statistical tests. No clear-cut distinction between true damage and nucleotide polymorphism can be obtained when only limited sequence coverage is available [23].

### Nucleotide Misincorporation Analysis and Identification of Contaminant Sequences

The misincorporation rates observed in both the ancient and modern datasets are higher than those expected on the basis of the error rates of the employed DNA polymerases. This result is not surprising in the case of aDNA as the theory predicts that nucleotide misincorporations can be produced by both damage and polymerase error. Such a discrepancy, reported by previous authors [22] is less obvious in the case of modern DNA. One potential explanation is that the error rates indicated by the companies for marketed enzymes are average values. It has been shown that the error rate of a certain DNA polymerase can vary according to the experimental conditions [17]. For example Gilbert et al. [22] reported an error rate of approximately 7×10^−4^ for the Platinum Taq Hifidelity (Invitrogen) polymerase while the error rate indicated by the producer is 2×10^−6^. A further cause of discrepancy may be the fact that modern DNA is subject to damage both in the extraction procedure and during PCR, while Lindahl's model [1] is largely based on *in vitro* induced damage.

The analysis of contaminants has been carried out on 44 clusters of Iceman's mtDNA sequences for which a distinction between endogenous and contaminant sequences had been previously established on a phylogenetical basis [24]. We can observe that the rate of misincorporation (*m*) for contaminant sequences is comparable to the rate found in the modern sequences. This result, although the underlying molecular mechanism is unclear, confirms the phylogenetical analysis.

The issue of distinguishing between original and contaminant sequences is crucial in ancient DNA studies and in particular in those dealing with human DNA. Contamination can arise at several stages during the taphonomical history of a specimen: during deposition or burial, during excavation, during storage at museums or during handling of the specimen when conducting morphological investigations. Exogenous DNA can also be introduced during extraction or amplification procedures [35]. Over the years, several guidelines have been proposed to reduce the impact of contamination during sample handling [36]–[40]. However, it is evident that a certain level of contamination is unavoidable and the main issue is to distinguish between original and contaminant sequences.

Our results suggest that the rate of misincorporation, which shows different values in the ancient and modern datasets, can be a practical parameter to deal with it. The only limitation to the approach based on the rate of misincorporation can be envisaged in the case of ancient contaminants, as they can be damaged to an extent comparable to original DNA. In particular, it has been shown that contaminants that are more than 10 years old have approximately five times more damage than those that are recent [41].

### Type 1 Versus Type 2 Transitions

The spectrum of nucleotide misincorporations (transitions and transversions) in the Iceman's dataset shows a strong dominance of transitions. This phenomenon has been previously reported for aDNA damage datasets. Within the transitions, we observe a strong bias towards type 2 transitions, with (following scaling) 635.07 type 1 and 5143.16 type 2 (∼1∶8 ratio) transitions. Our results are consistent with those of Hofreiter et al. [10], Stiller et al. [19], Brotherton et al. [21] and Gilbert et al. [22], who reported very strong biases toward type 2 transitions in the ancient sequences.

Type 2 transitions can be produced by hydrolytic deamination of cytosine and its homologue 5-methyl cytosine to U and T, respectively, which generate C→T substitutions during replication. It is true that, in principle, type 2 transitions could also be produced by modifications of G nucleotides which are then read as A. Yet several studies [19], [21], [22] have now conclusively shown that the incidence of G nucleotide modifications is negligible.

As for the origin of type 1 transitions, in the past there has been debate about the existence, or not, of some kind of damage on template DNA which could generate this type of misincorporation. However it has recently become clear that the relatively high incidence of type 1 transitions reported by some earlier PCR-based studies strongly decreases once alternative techniques such as high-throughput pyrosequencing on the 454 platform [19]–[22] are employed, thus making unlikely the damage hypothesis.

Our results show a clear-cut difference in the ratio type 1: type 2 transitions in ancient (1∶8) and modern (3∶1) sequences. A detailed analysis of the results shows that this is attributable to a strong decrease of type 2 transitions in the modern sequence dataset compared to the ancient one. In addition, we observe a slight increase of type 1 transitions in the modern sequence dataset (Figure 3), a phenomenon which further contributes to the inversion of the ratio compared to the ancient one.

We can now compare the present results, obtained with a mixed PCR-454 protocol with the results of investigations based on the exclusive use of the 454 platform [22] and with the results of a pure PCR system [18] (Table 3). To make them comparable, all the data have been scaled to 10^6^ nucleotides. We can observe that, indeed, the incidence of type 1 transitions is very low in the 454 platform system (respectively 296.64 and 199.51 for the ancient and the modern specimen) compared to both the pure PCR system (1990.26 for the ancient specimen) and the mixed PCR-454 system (1006.02 and 1415.88 respectively for the ancient and the modern specimen). This observation confirms the idea that type 1 transitions are mainly PCR artefacts. We also note that the incidence of type 2 transitions is much closer in the three groups of data (4540.82, 6386.07, and 4692.70 for the ancient specimens and 133.00 and 419.79 for the modern specimens respectively) than is that of type 1 transitions thus suggesting that all three techniques (454 platform, mixed PCR-454 platform, PCR) provide an acceptable estimate of aDNA damage. On the other hand, the prevailing source of damage seems to be cytosine deamination. In this sense our results support the validity of the idea of discriminating between ancient and contaminant DNA on the basis of the statistic of cytosine deaminations as proposed by Helgason and colleagues [42].

**Table 3:** Incidence of type 1 and type 2 transitions in ancient and modern DNA sequences obtained by PCR, 454 platform, or both.

	Type of Sample	Type 1 transition*	Type 2 transition*
**This study**	Ancient	1006.02	6386.07
	Modern	1415.88	419.79
**Gilbert et al. ** **[22]**	Ancient	296.64	4540.82
	Modern	199.51	133.00
**Binladen et al. ** **[18]**	Ancient	1990.26	4692.70

* The value of type 1 and type 2 transitions are scaled to 10^6^ nucleotides.

### Conclusions

The analysis of post-mortem damage in the Iceman's mitochondrial sequences produced by a mixed sequencing procedure based on PCR amplification and 454 sequencing contributes to the knowledge of nucleotide misincorporation patterns in ancient DNA sequences from frozen, freeze-dried and permafrost remains.

Our data contributes to the knowledge that the vast majority of ancient DNA damage is represented by type 2 transitions caused by cytosine deamination and that type 1 transitions could be attributable to PCR artefacts. The use of large sequence datasets has allowed us to distinguish, in a statistically significant way, between the mummy's original sequences and contaminants on the basis of the rate of nucleotide misincorporation which confirms a previous identification made on a purely phylogenetical basis. Our study also suggests that a distinction between original and contaminant sequences for ancient samples cannot be reliably performed when only small sequence datasets are available.

## Supporting Information

Table S1 Ancient sequences nucleotide misincorporation calculations and nucleotide misincorporation rate within each mitochondrial fragment.
(0.05 MB PDF)

Table S2 Modern sequences nucleotide misincorporation calculations and nucleotide misincorporation rate within each mitochondrial fragment.
(0.05 MB PDF)

Table S3 Contaminant sequences nucleotide misincorporation rate within each mitochondrial fragment.
(0.01 MB PDF)
