# Supplementary material for: Characterization of Nucleotide Misincorporation Patterns in the Iceman's Mitochondrial DNA
Source: PLoS One. 2010 Jan 8;5(1):e8629. doi: 10.1371/journal.pone.0008629 (PMC2799664; doi:10.1371/journal.pone.0008629)
Supplement: Table S1 — Ancient sequences nucleotide misincorporation calculations and nucleotide misincorporation rate within each mitochondrial fragment. (0.05 MB PDF) [file pone.0008629.s001.pdf]

Table S1. Ancient sequences nucleotide misincorporation calculations and nucleotide misincorporation rate within each mitochondrial fragment.

						Nucleotide misincorporation													Complementary Groups					
Mitochondrial fragment	Number of clones	Lenght of fragment	Number of nucleotide misincorporations	*m*	AT/GC Ratio	A→C	T→C	A→G	T→G	A→T	T→A	C→A	G→T	C→G	G→C	C→T	G→A	Total	A→G T→C	A→T T→A	A→C T→G	C→T G→A	C→G G→C	C→A G→T
L16518/H47	270	97	222	0.008477	1.16	0	6	22	0	0	0	0	1.16	0	0	198.76	24.27	252.19	28	0	0	223	0	1.16
L39/H118	108	78	86	0.010209	0.77	0	5.16	0	0	1.29	2.58	0	2	0	0	50	27	88.1	5.16	3.87	0	77	0	2
L207/H343	41	135	43	0.007769	1.45	1	1	0	0	0	1	1.45	0	0	0	56.73	0	61.18	1	1	1	56.73	0	1.45
L707/H809	220	101	181	0.008146	0.91	0	8.8	24.2	0	1.1	0	2	0	1	0	107	40	184.22	33	1.1	0	147	1	2
L1135/H1202	481	66	225	0.007088	0.94	3.18	13.78	13.78	2.12	0	1.06	11	4	0	0	102	76	227	27.56	1.06	5.3	178	0	15
L1170/H1211	134	40	48	0.008955	0.75	8.1	5.4	0	0	0	0	9	0	0	0	27	2	51.33	5.4	0	8.1	29	0	9
L1177/H1238	112	61	45	0.006587	0.97	1.03	1.03	5.15	0	0	1.03	2	0	0	0	30	5	45.26	6.18	1.03	1.03	35	0	2
L1361/H1493	19	131	10	0.004018	1.08	0	2	1	0	0	0	0	0	0	0	1.08	6.48	10.56	3	0	0	7.56	0	0
L1720/H1833	62	112	36	0.005184	1.8	0	2	2	0	1	0	5.4	3.6	0	0	27	19.8	60.8	4	1	0	46.8	0	9
L2643/H2746	76	112	52	0.006109	1.24	0	6	2	0	0	0	0	1.24	0	0	34.72	18.6	62.56	8	0	0	53.32	0	1.24
L3380/H3497	89	116	66	0.006393	1	0	0	2	0	0	0	0	0	0	0	48	16	66	2	0	0	64	0	0
L3477/H3530	165	52	47	0.005478	0.79	0	0	2.52	0	0	0	6	0	0	0	38	1	47.52	2.52	0	0	39	0	6
L4687/H4776	93	88	41	0.005010	2.14	0	1	6	0	0	0	0	0	0	0	70.71	2.14	79.85	7	0	0	72.85	0	0
L4730/H4849	131	119	153	0.009815	1.38	0	18	12	2	1	0	4.14	2.76	0	0	155.94	2.76	198.6	30	1	2	158.7	0	6.9
L6951/H7074	155	122	120	0.006346	1.3	5	9	16	0	2	1	0	1.3	0	0	89.83	22.13	146.26	25	3	5	112	0	1.3
L6999/H7107	93	107	106	0.010652	1.33	1	6	3	0	0	0	0	0	1.33	0	70.28	55.7	137.31	9	0	1	126	1.33	0
L8081/H8165	142	83	77	0.006533	1.13	0	4	4	0	0	0	0	5.64	0	2.26	40.62	29.33	85.85	8	0	0	69.95	2.26	5.64
L8776/H8872	21	95	9	0.004511	0.98	0	0	2.04	0	0	0	0	0	0	0	4	3	9.04	2.04	0	0	7	0	0
L8840H8932	59	91	54	0.010058	1.07	1	3	1	13	0	0	0	0	0	0	36.32	2.14	56.46	4	0	14	38.46	0	0
L9027/H9105	245	77	133	0.007050	1.33	0	15	5	0	0	0	1.33	0	0	0	144	5.33	170.66	20	0	0	149.3	0	1.33
L9612/H9715	100	102	49	0.004804	1.68	0	4	3	0	0	7	0	0	0	0	30.32	28.63	72.95	7	7	0	58.95	0	0
L9678/H9740	247	61	91	0.006040	1.77	0	5	8	0	0	0	0	1.77	0	0	108.14	28.36	151.27	13	0	0	136.5	0	1.77
L9696/H9785	151	88	85	0.006397	1.26	0	6	2	4	0	1	1.26	0	0	0	76.64	12.56	103.46	8	1	4	89.2	0	1.26
L10377/H10500	37	122	15	0.003323	2.7	0	1	5	0	0	0	0	0	0	0	16.18	8.09	30.27	6	0	0	24.27	0	0
L10476/H10599	57	122	42	0.006040	1.9	0	5	0	0	1	1	1.9	0	0	0	60.95	3.81	73.66	5	2	0	64.76	0	1.9
L10545/H10646	142	100	104	0.007324	1.62	0	3	10	0	0	1	0	0	0	0	129.52	16.19	159.71	13	1	0	145.7	0	0
L11261/H11325	293	63	213	0.011539	1.25	0	3	8	0	0	1	1.25	0	0	0	243.75	6.25	263.25	11	1	0	250	0	1.25
L11274/H11381	172	106	148	0.008118	1.72	0	14	9	0	0	4	0	0	1.72	0	175.23	30.92	234.87	23	4	0	206.2	1.72	0
L11408/H11536	60	108	42	0.006481	1.04	1	5	2	0	0	1	0	1.04	0	0	29.06	4.15	43.25	7	1	1	33.21	0	1.04
L11456/H11536	72	79	31	0.005450	1.19	0	0	2	0	0	1	0	2.39	0	0	27.47	3.58	36.44	2	1	0	31.05	0	2.39
L11620/H11742	72	122	42	0.004781	1.1	0	1	6	0	0	1	1.1	2.21	0	0	32	2.21	45.52	7	1	0	34.21	0	3.31
L11711/H11778	169	67	80	0.007065	1.09	1	1	12	0	0	0	1.09	0	0	0	62.34	8.75	86.18	13	0	1	71.09	0	1.09
L12233/H12315	79	81	31	0.004845	1.45	0	1	2	0	0	0	0	0	0	0	34.91	5.82	43.73	3	0	0	40.73	0	0
L12283/H12398	42	95	33	0.008271	1.44	1	5	2	0	0	0	0	0	0	0	34.46	1.44	43.9	7	0	1	35.9	0	0
L12370/H12449	111	78	78	0.009009	1.52	1	7	8	2	0	0	0	0	0	0	89.45	1.52	108.97	15	0	3	90.97	0	0
L14097H14204	1030	106	853	0.007813	1.65	6	66	60	2	1	4	34.65	3.3	1.65	0	1112.1	26.4	1317.1	126	5	8	1139	1.65	37.95
L14697/H14799	97	102	74	0.007479	1.91	1	7	12	0	0	0	0	1.91	0	0	95.71	5.74	123.36	19	0	1	101.5	0	1.91
L14752/H14869	118	116	91	0.006648	1.07	1	5	2	0	0	1	0	0	0	0	79.29	8.57	96.86	7	1	1	87.86	0	0
L15292/H15384	352	91	401	0.012519	1.02	2	12	25	0	0	2	0	3.07	0	1.02	349.6	14.31	409	37	2	2	363.9	1.02	3.07
L15320/H15418	103	97	118	0.011811	1.06	3	12	4	0	1	0	2.13	0	0	0	101.06	1.06	124.25	16	1	3	102.1	0	2.13
L16209/H16331	41	121	44	0.008869	1.16	0	1	5	0	0	0	1.16	0	0	0	42.95	0	50.11	6	0	0	42.95	0	1.16
L16303/H16407	161	103	132	0.007960	1.02	4	7	12	0	1	0	3.06	1.02	0	2.04	100.94	3.06	134.12	19	1	4	104	2.04	4.08
L16347/H16473	174	125	162	0.007448	0.67	0	9	6	0	0	0	0	2	0	0	128	22	167	15	0	0	150	0	2
L16429/H16537	123	107	69	0.005243	0.95	0	5.29	13.75	0	0	0	0	2	0	0	30	19	70.04	19.04	0	0	49	0	2

NOTE. — Mitochondrial fragments are numbered with reference to the Cambridge reference sequence (rCRS)[28].
