# Supplementary material for: Characterization of Nucleotide Misincorporation Patterns in the Iceman's Mitochondrial DNA
Source: PLoS One. 2010 Jan 8;5(1):e8629. doi: 10.1371/journal.pone.0008629 (PMC2799664; doi:10.1371/journal.pone.0008629)
Supplement: Table S2 — Modern sequences nucleotide misincorporation calculations and nucleotide misincorporation rate within each mitochondrial fragment. (0.05 MB PDF) [file pone.0008629.s002.pdf]

Table S2. Modern sequences nucleotide misincorporation calculations and nucleotide misincorporation rate within each mitochondrial fragment.

						Nucleotide misincorporation													Complementary Groups					
**Mitochondrial fragment**	**Number of clones**	**Lenght of fragment**	**Number of nucleotide misincorporations**	***m***	**AT/GC Ratio**	**A→C**	**T→C**	**A→G**	**T→G**	**A→T**	**T→A**	**C→A**	**G→T**	**C→G**	**G→C**	**C→T**	**G→A**	**Total**	**A→G T→C**	**A→T T→A**	**A→C T→G**	**C→T G→A**	**C→G G→C**	**C→A G→T**
L16518/H47	168	97	42	0.002577	1.20	2	12	22	0	3	3	1.20	0	0	0	0	2.40	45.60	34	6	2	2.40	0	1.20
L39/H118	158	78	29	0.002353	0.77	0	7.74	10.32	0	7.74	3.87	0	1	0	0	1	4	35.67	18.06	11.61	0	5	0	1
L207/H343	26	135	15	0.004274	1.45	1	6	4	1	0	0	0	0	0	0	1.45	2.90	16.35	10	0	2	4.35	0	0
L707/H809	105	101	28	0.002640	0.91	0	5.5	16.5	0	1.1	1.1	2	0	0	0	3	1	30.20	22	2.2	0	4	0	2
L1135/H1202	195	66	37	0.002875	1.00	1	7	19	0	1	5	1	0	0	0	3	1	38	26	6	1	4	0	1
L1170/H1211	339	40	45	0.003319	0.82	1.22	28.06	6.1	2.44	0	1.22	0	0	0	0	9	4	52.04	34.16	1.22	3.66	13	0	0
L1177/H1238	280	61	34	0.001991	1.03	1	8	10	1	0	0	1.03	0	1.03	0	9.30	3.10	34.46	18	0	2	12.4	1.03	1.03
L1361/H1493	149	131	36	0.001844	1.10	1	7	20	0	0	0	1.10	0	0	1.097	5.48	1.10	36.77	27	0	1	6.58	1.10	1.10
L1720/H1833	144	112	49	0.003038	1.87	2	7	24	0	3	0	0	0	0	0	16.85	7.49	60.34	31	3	2	24.33	0	0
L2643/H2746	52	112	20	0.003434	1.24	0	4	7	1	2	1	0	0	0	0	1.24	4.96	21.20	11	3	1	6.2	0	0
L3380/H3497	172	116	53	0.002656	1.04	3	7	23	0	4	3	1.04	0	1.04	0	5.18	6.21	53.47	30	7	3	11.39	1.04	1.04
L3477/H3530	364	52	36	0.001902	0.79	2.52	8.82	10.08	0	2.52	1.26	3	0	0	0	11	2	41.20	18.9	3.78	2.52	13	0	3
L4687/H4776	770	88	123	0.001815	2.14	4	37	43	0	10	7	2.14	0	0	0	32.14	12.9	148.18	80	17	4	45	0	2.14
L4730/H4849	41	119	12	0.002460	1.38	0	3	6	0	1	0	1.38	0	0	0	0	1.38	12.76	9	1	0	1.38	0	1.38
L6951/H7074	227	122	51	0.001842	1.30	1	24	8	1	4	11	0	0	0	0	0	2.60	51.60	32	15	2	2.604	0	0
L6999/H7107	253	107	55	0.002032	1.33	1	36	17	1	0	2	0	0	0	0	7.96	2.65	67.61	53	2	2	10.61	0	0
L8081/H8165	252	83	53	0.002534	1.08	4	10	21	1	1	4	3.23	0	0	0	13.98	2.15	60.36	31	5	5	16.13	0	3.23
L8776/H8872	155	95	22	0.001494	0.94	0	6.42	4.28	0	3.21	2.14	2	0	0	0	3	2	23.05	10.7	5.35	0	5	0	2
L8840H8932	223	91	60	0.002957	1.02	0	10	16	0	1	1	0	0	4.09	0	17.38	11.24	60.71	26	2	0	28.62	4.09	0
L9027/H9105	122	77	24	0.002555	1.26	1	9	3	0	1	3	2.53	1.26	0	0	5.06	0	25.85	12	4	1	5.06	0	3.79
L9612/H9715	184	102	35	0.001865	1.76	0	9	19	0	2	1	1.76	0	0	0	1.76	3.51	38.03	28	3	0	5.27	0	1.76
L9678/H9740	404	61	51	0.002069	1.90	0	20	16	2	1	4	1.90	0	1.9	0	5.71	5.71	58.22	36	5	2	11.43	1.90	1.90
L9696/H9785	185	88	32	0.001966	1.32	0	17	8	0	0	2	2.63	0	0	0	2.63	1.31	33.57	25	2	0	3.95	0	2.63
L10377/H10500	88	122	23	0.002142	2.81	0	6	8	1	4	0	0	0	0	0	5.63	5.63	30.26	14	4	1	11.25	0	0
L10476/H10599	193	122	48	0.002039	1.98	0	23	19	1	0	2	0	0	0	0	3.95	1.98	50.93	42	2	1	5.93	0	0
L10545/H10646	384	100	74	0.001927	1.44	1	21	21	3	7	5	1.44	0	0	0	14.39	7.20	81.03	42	12	4	21.59	0	1.44
L11261/H11325	450	63	49	0.001728	1.33	0	12	22	1	3	2	1.33	1.33	0	1.33	5.33	2.67	51.99	34	5	1	8	1.33	2.67
L11274/H11381	336	106	96	0.002695	1.79	1	26	34	3	8	7	3.58	0	1.79	0	12.53	12.53	109.43	60	15	4	25.05	1.79	3.58
L11408/H11536	68	108	8	0.001089	1.08	0	3	3	0	0	0	1.08	0	0	0	1.08	0	8.16	6	0	0	1.08	0	1.08
L11456/H11536	142	79	34	0.003031	1.26	0	6	19	0	1	3	1.26	0	0	0	2.51	2.51	35.28	25	4	0	5.03	0	1.26
L11620/H11742	117	122	21	0.001471	1.07	0	7	7	0	0	1	0	0	0	0	4.27	2.14	21.41	14	1	0	6.41	0	0
L11711/H11778	352	67	59	0.002502	1.03	1	20	21	1	0	5	0	0	1.03	0	10.30	6.18	65.51	41	5	2	16.49	1.03	0
L12233/H12315	134	81	21	0.001935	1.53	3	4	9	0	1	2	0	0	0	0	1.53	1.53	22.06	13	3	3	3.06	0	0
L12283/H12398	130	95	27	0.002186	1.44	1	9	6	0	2	2	2.87	0	0	0	7.18	0	30.05	15	4	1	7.18	0	2.87
L12370/H12449	52	78	17	0.004191	1.44	0	5	6	1	1	0	0	0	0	0	5.75	0	18.75	11	1	1	5.75	0	0
L14097H14204	170	106	36	0.001998	1.59	3	15	12	2	2	0	0	0	0	0	12.68	0	46.68	27	2	5	12.68	0	0
L14697/H14799	238	102	80	0.003295	1.91	7	16	37	1	3	2	3.83	0	1.91	1.914	13.40	5.74	92.79	53	5	8	19.14	3.83	3.83
L14752/H14869	227	116	45	0.001709	1.07	1	14	14	0	2	0	2.14	0	0	0	10.71	2.14	45.99	28	2	1	12.86	0	2.14
L15292/H15384	183	91	40	0.002402	1.02	1	10	15	1	3	1	0	0	1.02	1.022	3.07	4.09	40.20	25	4	2	7.16	2.04	0
L15320/H15418	245	97	41	0.001725	1.06	4	9	13	0	0	3	1.06	0	0	0	10.64	1.06	41.76	22	3	4	11.70	0	1.06
L16209/H16331	136	121	47	0.002856	1.16	1	9	19	0	4	3	2.32	0	3.48	0	5.80	1.16	48.76	28	7	1	6.96	3.48	2.32
L16303/H16407	204	103	45	0.002142	1.10	0	11	21	0	2	1	0	1.10	0	0	6.61	3.31	46.02	32	3	0	9.92	0	1.1
L16347/H16473	91	125	22	0.001934	0.76	0	13.1	7.86	0	2.62	0	0	0	0	0	3	1	27.58	20.96	2.62	0	4	0	0
L16429/H16537	74	107	15	0.001894	1.02	0	4	5	0	1	1	0	0	0	0	3.06	1.02	15.08	9	2	0	4.08	0	0

NOTE. — Mitochondrial fragments are numbered with reference to the Cambridge reference sequence (rCRS)[28].
