# Supplementary material for: Characterization of Nucleotide Misincorporation Patterns in the Iceman's Mitochondrial DNA
Source: PLoS One. 2010 Jan 8;5(1):e8629. doi: 10.1371/journal.pone.0008629 (PMC2799664; doi:10.1371/journal.pone.0008629)
Supplement: Table S3 — Contaminant sequences nucleotide misincorporation rate within each mitochondrial fragment. (0.01 MB PDF) [file pone.0008629.s003.pdf]

Table S3. Contaminant sequences nucleotide misincorporation rate within each mitochondrial fragment.

Mitochondrial fragment	Number of clones	Lenght of fragment	Number of nucleotide misincorporations	*m*
L16518/H47	19	97	5	0.00271
L1177/H1238	14	61	3	0.00351
L1720/H1833	14	112	0	0
L11261/H11325	13	63	8	0.00977
L11456/H11536	29	79	0	0
L14097/H14204	22	106	5	0.00214
L16303/H16407	29	103	6	0.00201

NOTE. — Mitochondrial fragments are numbered with reference to the Cambridge reference sequence (rCRS) [28].
